# Circular RNA–microRNA–mRNA network identified circ_0007618 and circ_0029426 as new valuable biomarkers for lung adenocarcinoma

**DOI:** 10.1080/21655979.2022.2027180

**Published:** 2022-02-25

**Authors:** Qiang Zhang, Yungang Sun, Chao Wang, Feng Shao

**Affiliations:** a Department of Thoracic Surgery, Nanjing Chest Hospital, Nanjing, China; b Department of Thoracic Surgery, Affiliated Nanjing Brain Hospital, Nanjing Medical University, Nanjing, China; c Department of Thoracic Surgery, Pulmonary Nodule Diagnosis and Treatment Research Center, Nanjing Medical University, Nanjing, China

**Keywords:** Lung adenocarcinoma, circ_0007618, circ_0029426, diagnosis, prognosis

## Abstract

Circular RNAs (circRNAs) are involved in multiple physiological processes. However, whether circRNAs function as the indicators of lung adenocarcinoma (LUAD) remains unclear. Three LUAD-related microarray datasets were downloaded from the Gene Expression Omnibus database, and overlapping differentially expressed circRNAs (DECs) in LUAD were identified. circ_0007618 and circ_0029426 were revealed to be significantly dysregulated in LUAD and verified in LUAD tissues and serum obtained in this study. Subsequently, the overall survival curve and receiver operating characteristics curve analyses were performed to evaluate the prognosis, sensitivity, and specificity of circ_0007618 and circ_0029426 for LUAD diagnosis. The results indicate that the combination of circ_0007618 and circ_0029426 is a potential biomarker for LUAD diagnosis and prognosis. TargetScan and miRDB were used to predict interactions between microRNAs (miRNAs) and circRNAs/mRNAs. A circRNA–miRNA–mRNA network was established for LUAD diagnosis. The Kyoto Encyclopedia of Genes and Genomes and protein–protein interaction network identified four hub genes. In conclusion, circ_0007618 and circ_0029426 may be novel biomarkers for LUAD diagnosis and prognosis. For LUAD diagnosis, PIK3CA and NRAS, and KRAS and ETS1, were targeted by circ_0007618 and circ_0029426, respectively.

## Introduction

Lung adenocarcinoma (LUAD) is the most common histological type of lung cancer, with a high mortality rate and a low five-year survival rate [1]. Despite the rapid progress in clinical medicine and experimental oncology related to lung cancer, there remain no reliable indicators for the prognosis and early diagnosis of LUAD [2]. Elucidating the mechanism of the occurrence and development of LUAD will help improve LUAD diagnosis and treatment.

Accumulating evidence has clarified the molecular mechanism of LUAD via circular RNA (circRNA) [3,4]. Circular RNAs are a sub-type of endogenous non-coding RNAs with superior stability and can regulate gene expression in eukaryotes [5,6]. The circRNAs are stably present in body fluids and tissues of patients with diverse tumors, including lung, gastric, and breast cancers and glioma [7–10], demonstrating that they have the potential to function as novel noninvasive biomarkers. For instance, upregulation of circ-CAMK2A was observed in LUAD and linked to metastasis progression [11]. Likewise, dysregulation of circRNA_002178, circXPO1, and circDCUN1D4 was also identified in LUAD [3,12,13], implying that circRNAs play important roles in LUAD pathogenesis. Therefore, we identified novel circRNAs for the early diagnosis and prognosis of LUAD due to the vacancy of effective circRNAs for LUAD.

In this study, we aimed to identify aberrant expressed circRNAs in LUAD through bioinformatic analyze, and study the potential for LUAD diagnosis and prognosis. Furthermore, circRNA-miRNA-mRNA interaction analysis and protein-protein interaction (PPI) network were constructed to visualize the interactions downstream mRNAs.

## Materials and methods

### Clinical sample collection

We included 120 LUAD patients and 80 healthy volunteers archiving in Nanjing ChestHospital in this study, and 5 mL blood samples were collected from all patients. A total of 120 LUAD specimens and 80 adjacent normal tissue samples were collected. None of the patients received any chemotherapy or radiotherapy prior to surgery. Clinical data of patients were collected, including age, sex, tumor size, tumor stage, lymph node metastasis, carcinoembryonic antigen (CEA), and epidermal growth factor receptor (EGFR) mutation; the clinical information of all patients is shown in Tables 1 and 2. All the LUAD specimens were confirmed by two pathologists. The patients were followed via telephone. The time of operation was considered the initial time of follow-up, and overall survival (OS) was defined as death due to LUAD. All procedures were performed after the patients signed an informed consent form (ethical batch number: EC201500317-5). The tissue samples and serum obtained after centrifugation were stored at −80°C for subsequent experiments.

**Table 1.** Association between the circRNA expression levels in tumor tissues and clinicopathological characteristics of LUAD patients

Clinicopathologic characteristics	*n*	hsa_circ_0007618			hsa_circ_0029426		
		Low (n = 47)	High (n = 73)	*p*-value	Low (n = 69)	High (n = 51)	*p*-value
Age (years)				0.6761			0.2798
< 60	52	19	33		27	25	
≥ 60	68	27	40		42	26	
Sex				0.4303			0.4434
Male	61	26	35		33	28	
Female	59	21	38		36	23	
Smoking				0.0643			0.3008
No	76	25	51		41	35	
Yes	44	22	22		28	16	
Tumor size				0.5628			0.8168
<30 mm	65	27	38		38	27	
≥30 mm	55	20	35		31	24	
T stage				0.0100*			0.0247*
I–II	54	28	26		25	29	
III–IV	66	19	47		44	22	
Lymphatic metastasis				0.0471*			0.0348*
Negative	72	23	49		47	25	
Positive	48	24	24		22	26	
carcinoembryonic antigen (CEA)				0.1274			0.0318*
Negative	56	26	30		38	18	
Positive	64	21	43		31	33	
epidermal growth factor receptor (EGFR) mutation				0.0227*			0.0275*
Negative	43	11	32		19	24	
Positive	77	36	41		50	27	

*P < 0.05.

**Table 2.** Association between the serum circRNA expression levels and clinicopathological characteristics of LUAD patients

Clinicopathologic characteristics	n	hsa_circ_0007618			hsa_circ_0029426		
		Low (n = 53)	High (n = 67)	p-value	Low (n = 64)	High (n = 56)	*p*-value
Age (years)				0.8289			0.5604
< 60	44	20	24		25	19	
≥ 60	76	33	43		39	37	
Sex				0.7938			0.9026
Male	65	28	37		35	30	
Female	55	25	30		29	26	
53Smoking				0.1637			0.3532
No	74	29	45		37	37	
Yes	46	24	22		27	19	
Tumor size				0.2298			0.3315
<30 mm	72	35	37		41	31	
≥30 mm	48	18	30		23	25	
T stage				0.0274*			0.0354*
I–II	50	28	22		21	29	
III–IV	70	25	45		43	27	
Lymphatic metastasis				0.0418*			0.0217*
Negative	69	25	44		43	26	
Positive	51	28	23		21	30	
CEA				0.8205			0.0636
Negative	58	25	33		36	22	
Positive	62	28	34		28	34	
EGFR mutation				0.0257*			0.0233*
Negative	45	14	31		18	27	
Positive	75	39	36		46	29	

*P < 0.05.

### Cell culture

Human lung cancer cell lines (PC9, H460, A549 and H1299) and normal bronchial epithelial cells (BEAS-2B) were obtained from Shanghai Institute of Biochemistry and Cell Biology (Shanghai, China). Mixture of Dulbecco’s modified Eagle’s medium (Gibco, Grand Island, NY, USA), 10% fetal bovine serum, 100 U/mL penicillin and 100 μg/mL streptomycin (Gibco) was applied for culture of lung cancer cell lines. BEAS-2B cell incubation was conducted in LHC-9 complete medium (Gibco) [14]. All cells were maintained at 37°C under 5% CO2.

### RNA extraction

Total RNA of tissues and cells, and RNA from cytoplasm and nucleus were obtained using a TRIzol kit (Invitrogen, MA, USA) according to the manufacturer’s protocol. As for separation of the nucleus from cytoplasm, accutase (STEMCELL Technologies, Shanghai, China) was used to clone and digest the cells into single cells. 10% input was absorbed and centrifuged at 500 g to obtain cell precipitation. A NE-PER™ Nuclear and Cytoplasmic Extraction Kit (Thermo Fisher, Shanghai, China) was used to add appropriate amount of pre-cooled CER1 solvent and pre-cooled CER2 reagent according to the cell volume, and then the supernatant was obtained as cytoplasmic component [15]. An appropriate amount of pre-cooled NER was added to the rest of the precipitate, and the supernatant obtained by centrifugation 21,000 g after ice incubation was the nuclear component.

### Genomic DNA (gDNA) extraction

Biotinylated DNA probes complementary to circ_0007618 and circ_0029426 were synthesized by Invitrogen (MA, USA) and dissolved in 500 mL of wash and binding buffer (0.5 M NaCl, 20 mM Tris-HCl, pH 7.5, 1 mM EDTA) [16]. A549 cell lysates were incubated with probe-coated beads at 25°C for 1.5 h, and after washing with the wash and binding buffer, the RNA complexes bound to the beads were eluted and extracted for real-time quantitative PCR (RT-qPCR) analysis.

### RT-qPCR

mRNA levels of circRNAs in tissues, serum samples, and reference gene GAPDH were evaluated by RT-qPCR using a RT Kit (TakaRa, Shiga, Japan) and PCR Master Mix (TakaRa) on an ABI PRISM7500 system (Bio-Rad, CA, USA), according to the manufacturer’s instructions. Briefly, 500 ng of RNA was reverse-transcribed into cDNA with random primers at a total volume of 20 μL. After RNA reverse transcription, quantitative PCR was performed with GAPDH as the general reference and cytoplasmic reference and U6 as nuclear reference [17]. The relative abundance of target gene was analyzed by 2^−ΔΔCt^ relative quantitative method. The reactions were initiated at 95°C for 30s, followed by 40 cycles of 95°C for 5 s and 60°C for 20s. This method was repeated three times.

### CircRNA expression profile analysis

Three LUAD-related circRNA expression microarray datasets, GSE158695 (data from three pairs of human non-small cell lung cancer [NSCLC] tissues and corresponding non-cancerous tissues), GSE101684 (data from four pairs of tumor samples and paired adjacent normal tissues at early stage LUAD), and GSE112214 (data from three pairs of human NSCLC lung samples and three matched adjacent normal samples), were downloaded from the Gene Expression Omnibus database. Raw data were reorganized as a raw count expression matrix, which was then normalized using the limma R package (Version 3.26.9) [18]. Afterward, the fold change and Student’s t-test were used to identify differentially expressed circRNAs (DECs) in the three profiles. The threshold for DEC screening was |Log2 (fold change)|≥ 2.0, and *p* < 0.05. Furthermore, graphical heatmaps and Venn diagrams were generated based on the circRNA information.

### Actinomycin D

To block transcription, 2 mg/mL actinomycin D (Sigma-Aldrich, MO, USA) was added to the cell culture medium of A549 cells, and dimethyl sulfoxide was used as a negative control [19]. After treatment with actinomycin D, A549 cells were subjected to real-time quantitative PCR (RT-qPCR). Each experiment was repeated three times.

### RNase R treatment

3 U/μg RNase R (Epicenter Technologies, WI, USA) was added to total RNA (2 μg) and incubated at 37°C for 1 h [19]. After treated with RNase R, A549 cells were subjected to RT-qPCR. Each experiment was repeated three times.

### Fluorescence in situ hybridization (FISH)

After digestion, A549 cells were incubated at 37°C for 1 hour with hybridization fluid of circRNA-Probe (8 ng/μL). After hybridization at 37°C overnight, the cells were washed, followed by adding DAPI dye solution, and incubated at dark for 8 min. After rinsing, anti-fluorescence quenching agent was dropped [20]. Images were observed and collected under nikon ECLIPSE CI fluorescence microscope (Tokyo, Japan). Each experiment was repeated three times.

### CircRNA–miRNA–mRNA interaction analysis

Circular RNA Interactome (https://circinteractome.irp.nia.nih.gov/) was used to illustrate circularization mechanism of circRNAs. The circRNA and miRNA interactions were predicted using TargetScan (http://www.targetscan.org/mmu_72/) and miRDB (http://www.mirdb.org/). The circRNA–miRNA–mRNA network was constructed using Cytoscape (https://cytoscape.org/) [21]. Meanwhile, Kyoto Encyclopedia of Genes and Genomes (KEGG) pathway enrichment analysis for target genes of miRNAs was performed using the clusterProfiler package (Version 2.4.3).

### Construction of a protein–protein interaction (PPI) network

The STRING (http://www.string-db.org/) database and Cytoscape were used to construct the PPI network, as previously described [22].

### Statistical analysis

Data were analyzed using GraphPad (Version 6.0; GraphPad Software, Inc., CA, USA) and expressed as the mean ± standard deviation. Differences were analyzed using the Student’s t-test and one-way analysis of variance. Survival analysis was carried out by Kaplan–Meier analysis, and the potential diagnostic value of circRNAs in LUAD was presented by receiver operating characteristic (ROC) curve analysis. Statistical significance was set at *p* < 0.05.

## Results

### Analyses of DECs in LUAD

**Figure 1.**
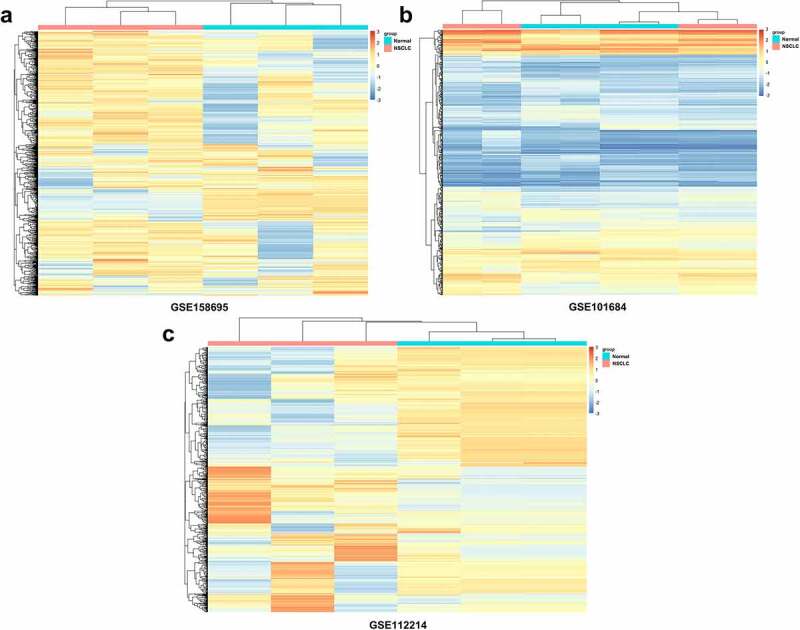
Heatmaps for differently expressed circRNAs (DECs) related to lung adenocarcinoma (LUAD) in three microarray datasets. Top color represents grouping, red: disease group, blue: control group; Orange in the heatmap: upregulated expression, blue: downregulated expression.

**Figure 2.**
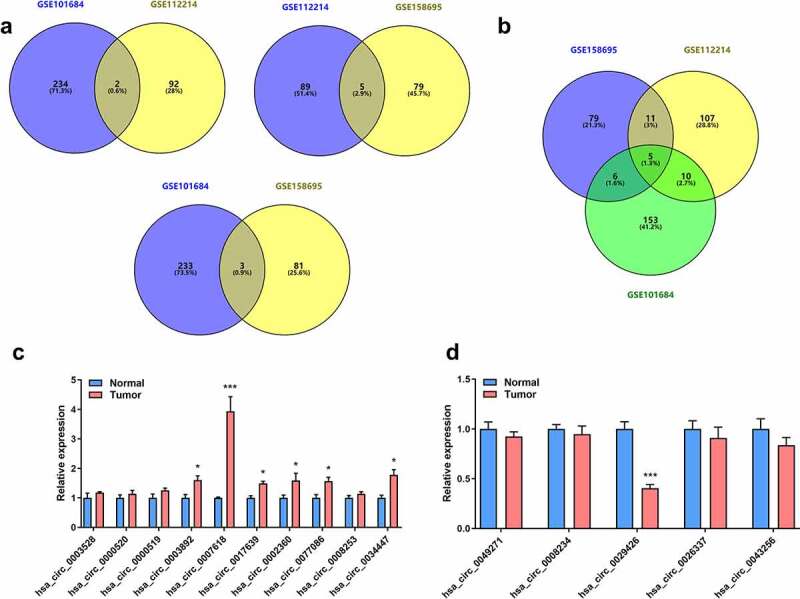
Venn diagram and expression of overlapping DECs in LUAD. (a) Venn diagram of overlapping upregulated circRNAs in LUAD. (b) Venn diagram of overlapping downregulated circRNAs in LUAD. (c, d) Expression of 15 selected circRNAs in clinical tumor samples. *p < 0.05, ***p < 0.001, compared with the normal group.

Using circRNA microarray datasets, a total of 872 circRNAs were obtained. In the GSE158695 group, 185 dysregulated circRNAs were identified, while the number of DECs in the GSE101684 and GSE112214 groups were 410 and 277, respectively. The heatmaps are shown in Figure 1. Furthermore, there were 10 upregulated and 5 suppressed candidates (Figure 2(a,b)). Next, 15 screened circRNAs were evaluated in LUAD tumor tissues and in normal controls. PCR analyses indicated that circ_0007618 was upregulated, while circ_0029426 was markedly downregulated in LUAD (Figure 2(c,d)).

### Circ_0007618 and circ_0029426 exist in lung cancer cells

**Figure 3.**
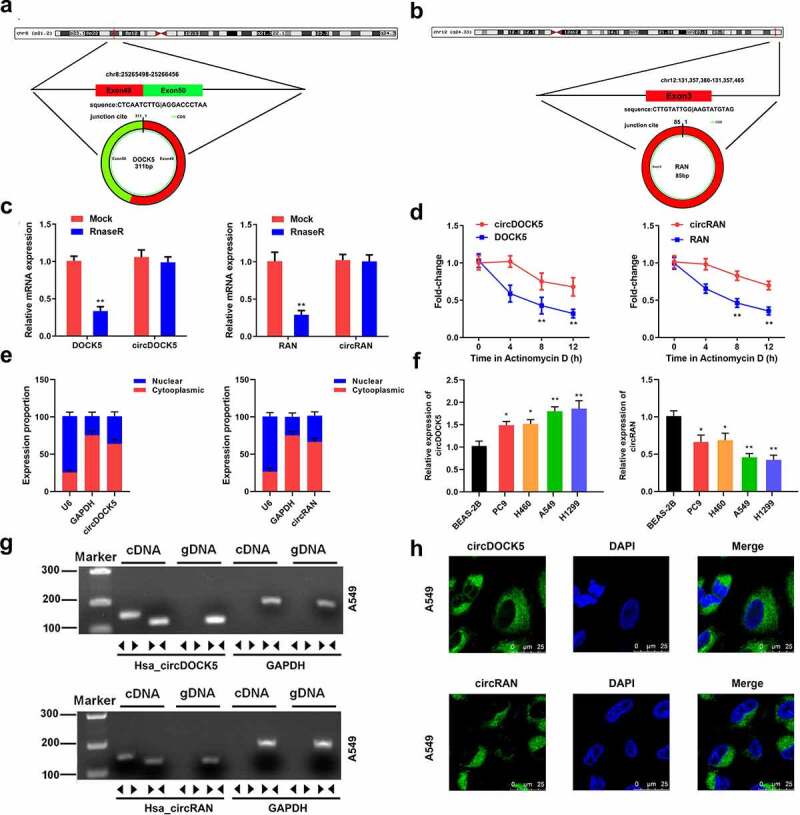
Circ_0007618 and circ_0029426 exist in lung cancer cells. (a, b) CircRNA circularization mechanism of circ_0007618 and circ_0029426. (c) The lung cancer cells were treated with RNase R and the expression of linear and circRNAs were detected by qPCR. (d) After actinomycin treatment, the half-lives of linear and circRNAs were detected by qPCR. (e) qPCR was used to detect the expression of circ_0007618 and circ_0029426 in the cell cytoplasm and nucleus. (f) qPCR was used to detect the expression of circ_0007618 and circ_0029426 in lung cancer cells. (g) Primers were designed, and PCR was performed in cDNA and gDNA and gelled for verification. (h) The FISH assay was used to detect the location of circ_0007618 and circ_0029426. **p < 0.01 vs Mock, circDOCK5, circRAN or BEAS-2B groups.

Next, wheather circ_0007618 and circ_0029426 exist in lung cancer cells was studied. CircRNA circularization mechanism showed that circ_0007618 was spliced by exons 49–50 of the DOCK5 transcript, and circ_0029426 was spliced by exon 3 of the RAN transcript (Figure 3(a,b)). Following RNase R treatment, linear and circular RNA expressions were tested. The results showed that the expression levels of circular DOCK5 and RAN were unchanged, while the linear DOCK5 and RAN levels were significantly reduced (Figure 3(c)). Following actinomycin treatment, linear and circular RNA half-life changes were detected. The results showed that circular DOCK5 and RAN had higher stability and longer half-lives (Figure 3(d)). RNA in the cell plasma and nucleus was separated, and the expression levels of circular DOCK5 and RAN in the plasma were considerably higher than those in the nucleus (Figure 3(e)). Consistently, the FISH assay revealed that circDOCK5 and circRAN were primarily located in the cytoplasm (Figure 3(f)). Divergent and convergent primers were designed, and PCR was performed. These results confirmed our previous findings. CircCDOCK5 and circRAN exist at the RNA level, however, not at the DNA level (Figure 3(g)). Moreover, we evaluated the expression of both circCDOCK5 and circRAN in lung cancer cells and found that circCDOCK5 was upregulated, while circRAN was downregulated (Figure 3(h)). These results indicated that circ_0007618 and circ_0029426 were present in lung cancer cells.

### Dysregulated circ_0007618 and circ_0029426 are associated with clinicopathological characteristics of LUAD

**Figure 4.**
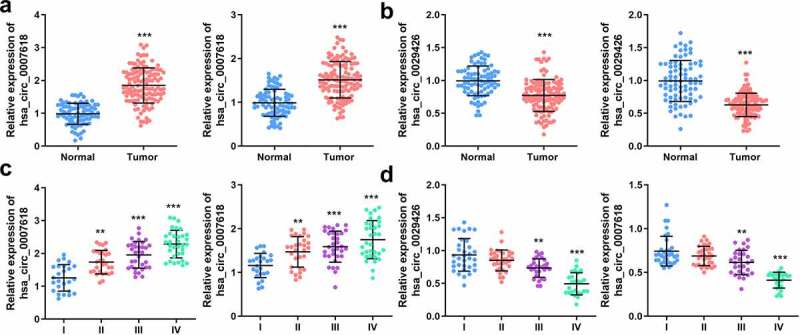
Expression of circ_0007618 and circ_0029426. (a, b) PCR analyses of circ_0007618 and circ_0029426 levels in tissues and serum samples of LUAD. (c, d) Expression of circ_0007618 and circ_0029426 in tissues and serum samples at different tumor stages. **p < 0.01, ***p < 0.001, compared with the normal and I groups.

Next, the mRNA levels of both circ_0007618 and circ_0029426 in tissues and serum samples of LUAD patients were detected by PCR, and the results revealed that circ_0007618 expression was significantly increased in both tumor tissues and serum, while circ_0029426 expression was significantly decreased in both samples (Figure 4(a,b)). Interestingly, abnormal circ_0007618 and circ_0029426 expression was significantly correlated with tumor stage, lymphatic metastasis, and EGFR mutation (*p* < 0.05; Table 1). circ_0029426 inhibition in tumor tissues and serum was also linked to the abovementioned clinicopathological characteristics; CEA level was associated with circ_0029426 expression in tumor tissues (*p* < 0.05). Furthermore, dysregulated circ_0007618 and circ_0029426 were significantly correlated with tumor stage (Figure 4(c,d)).

### Circ_0007618 and circ_0029426 may function as prognostic and diagnostic biomarkers of LUAD

**Figure 5.**
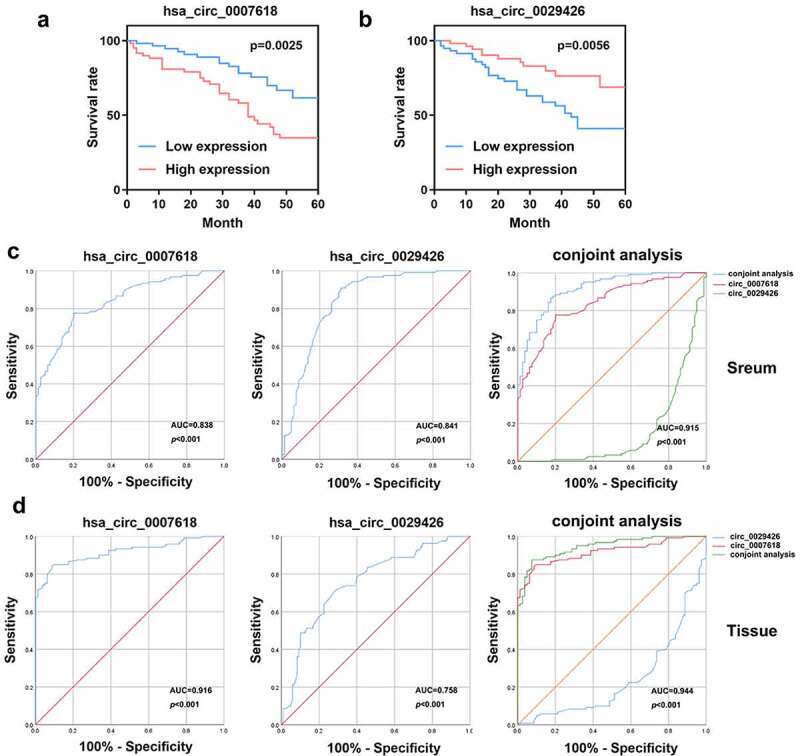
Overall survival (OS) curve and receiver operating characteristic (ROC) curves were used to evaluate the sensitivity and specificity of circ_0007618 and circ_0029426 for LUAD diagnosis.

We analyzed the OS of LUAD patients. As shown in Figure 5(a,b), high levels of circ_0007618 or circ_0029426 are associated with poor OS in LUAD patients. Moreover, ROC curve analysis suggested that the area under the curve (AUC) of circ_0007618 in tissue and serum were 0.916 (95% CI, 0.878–0.955) and 0.838 (95% CI, 0.784–0.892), respectively, while the AUC of circ_0029426 in tissue and serum was 0.758 (95% CI, 0.689–0.862) and 0.841 (95% CI, 0.780–0.901), respectively. Furthermore, the AUC of the conjoint analysis of circ_0007618 and circ_0029426 in tissue and serum were 0.944 (95% CI, 0.916–0.973) and 0.915 (95% CI, 0.878–0.953), respectively (Figure 5(c,d)). Therefore, circ_0007618 and circ_0029426 may be sensitive biomarkers for LUAD prognosis and diagnosis.

### Construction of circRNA–miRNA–mRNA network

**Figure 6.**
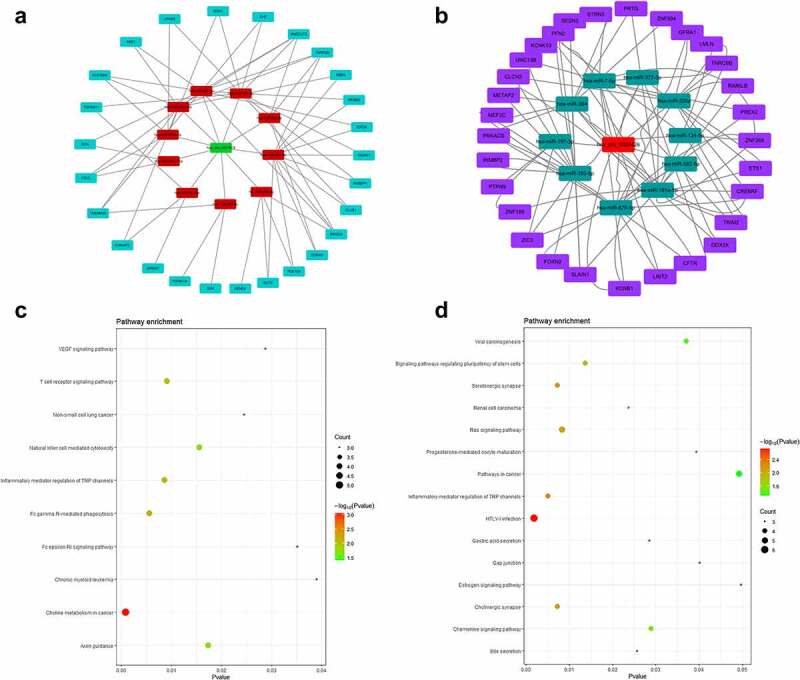
ceRNA network (a) and KEGG analyses (c) of circ_0007618 and circ_0029426 (b, d).

Then, we selected the intersection of miRNAs predicted in TargetScan and miRDB, and obtained 10 miRNAs (miR-627-3p, miR-597-3p, miR-676-5p, miR-224-3p, miR-26b-3p, miR-671-3p, miR-541-3p, miR-92a-1-5p, miR-514a-5p, and miR-302a-5p) that had binding sites with circ_0007618, 10 miRNAs (miR-7-5p, miR-377-3p, miR-320a, miR-134-5p, miR-382-5p, miR-181a-5p, miR-876-5p, miR-183-5p, miR-197-3p, and miR-384) for circ_0029426. Afterward, the ceRNA network was constructed by circ_0007618/circ_0029426, 10 screened miRNAs and 100 downstream genes (Figure 6(a,b)). KEGG analysis (corrected *p*-value < 0.05) demonstrated that the downstream genes of circ_0007618 were signifcantly enriched in 10 pathways, among which choline metabolism enriched the most genes. Meanwhile, the downstream genes of circ_0029426 were predominantly enriched in 15 pathways, among which HTLV-I infection and Ras signaling pathways enriched the most genes (Figure 6(c,d)).

### Identification of four hub genes from the PPI network

**Figure 7.**
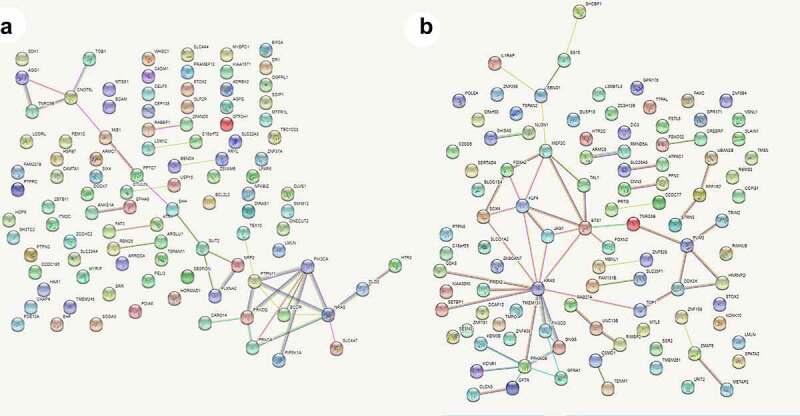
Protein–protein interaction (PPI) networks of circ_0007618 (a) and circ_0029426 (b).

**Figure 8.**
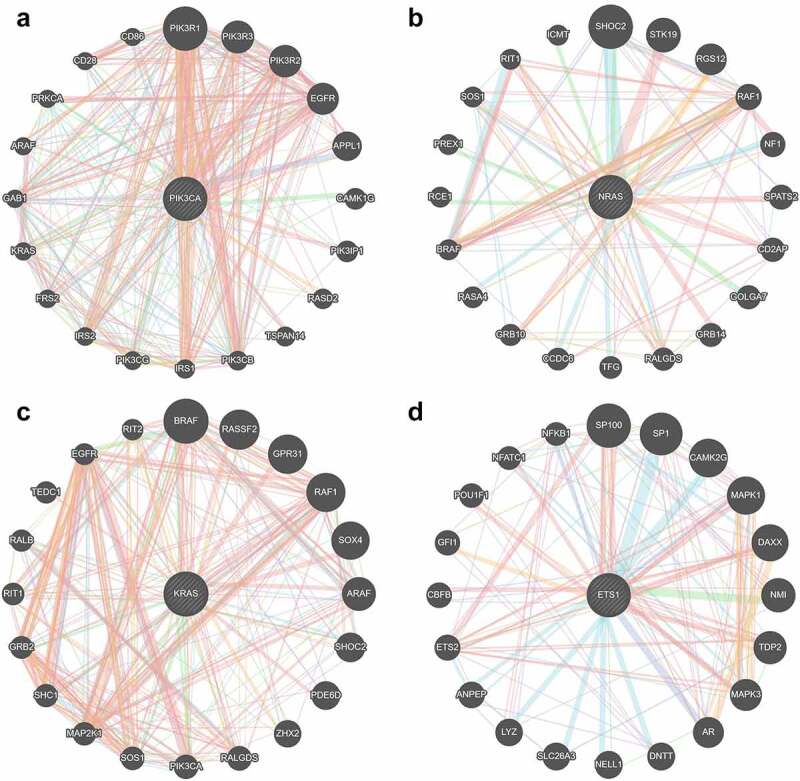
Hub genes related to circ_0007618 (a, b) and circ_0029426 (c, d).

A PPI network was constructed based on the interactions retrieved from STRING. The PPI network showed that 100 nodes and 86 protein pairs were included in the circ_0007618 network, and 100 nodes and 133 protein pairs were included in the circ_0029426 network (Figure 7(a,b)). PIK3CA and NRAS were identified as hub genes related to circ_0007618, and KARS and ETS1 were identified as hub genes related to circ_0029426 (Figure 8(a–d)).

## Discussion

LUAD is a common lung malignancy [1]. Currently, the treatment of LUAD is mainly surgery and chemotherapy, but the prognosis and diagnosis of LUAD is still not ideal [2]. Accumulating evidence suggested that circRNAs may be a new strategy for LUAD due to their stable structure and biological regulatory functions [5,6]. A related study found that circRNA ENO1 promoted aggressiveness of LUAD cells through upregulating its host gene ENO1 [4]. Dysregulated circRNA_002178 elevated PDL1 expression by sponging miR-34 in LUAD cells; furthermore, circRNA_002178 has been verified be a potential biomarker for LUAD early diagnosis via detection exosomes derived from LUAD patients [3]. These studies demonstrated that circRNAs may be a breakthrough point for LUAD prognosis and diagnosis.

Limited by small sample size or lack of relevant clinical data, it is not feasible to construct diagnostic or prognostic features for clinical application based on circRNA at this stage. However, a large amount of transcriptome data with relevant clinical data provides us with the possibility to construct clinically usable diagnostic or prognostic features. In the current study, 15 dysregulated circRNA candidates were screened from 872 identified DECs in LUAD. PCR analyses of circRNA expression levels in tumor tissues of LUAD patients revealed that circ_0007618 was markedly increased in LUAD, while circ_0029426 was markedly decreased. Hence, we speculated that circ_0007618 and circ_0029426 may be associated with LUAD progression. Furthermore, while the expression trends of circ_0007618 and circ_0029426 in serum were consistent with those in tumor tissues, the expression levels of both circRNAs in LUAD tissues and plasma were correlated with clinicopathological characteristics such as tumor stage, lymphatic metastasis, and EGFR mutation. The OS and ROC curves of both circ_0007618 and circ_0029426 in tissues and serum of LUAD patients suggested that evaluating circ_0007618 and circ_0029426 could provide sensitive biomarkers for LUAD prognosis and diagnosis. Considering the convenience of clinical sampling, detection of circ_0007618 and circ_0029426 in serum is more suitable for LUAD diagnosis and prognosis.

A previous study revealed that circ_0029426 may be a diagnostic indicator of glioblastoma [23] and can predict poor glioblastoma prognosis [24]. However, to date, the underlying mechanisms of circ_0007618 and circ_0029426 remain unclear. The circRNA–miRNA–mRNA axis was recently discovered to regulate the development of diseases [25] including LUAD and provided a new strategy for LUAD diagnosis and prognosis [26,27]. Our data showed that circ_0007618 and circ_0029426 were associated with LUAD and could bind several miRNAs to regulate downstream genes, among which several genes, such as PIK3CA and KARS, were regulated by multiple miRNAs. KEGG analyses further confirmed that the enrichment of these downstream genes was associated with choline metabolism and HTLV-I infection signaling pathways, which may induce cancer [28–31], indicating that circ_0007618 and circ_0029426 may regulate these cancer-related genes to regulate LUAD. The PPI network combined with KEGG analyses suggested that PIK3CA and NRAS were two hub genes regulated by circ_0007618, while KRAS and ETS1 were two hub genes for circ_0029426 in LUAD, which was partly in line with previous studies [32,33].

PIK3CA is a key enzyme in the PI3K signaling pathway that regulates the cellular functions of tumor cells [34]. PIK3CA mutations exist in numerous tumors, and LUAD patients with mutated PIK3CA exhibited significantly downregulated PD-L1 expression, affecting the efficacy of adjuvant chemotherapy [35]. The rat sarcoma (RAS) gene is the most frequently activated proto-oncogene in human cancers and is widely present in eukaryotic cells [36]. NRAS and KRAS are major members of the RAS gene family in mammals, and the proto-oncogene NRAS is abnormally highly expressed in various human malignant tumor tissues and corresponding cell lines, which can promote tumor cell invasion and metastasis [37,38]. KRAS is the most frequently mutated gene, followed by NRAS [39]. LUAD with NRAS and KRAS mutations are relatively common in clinical practice; there is currently no effective targeted therapy, and its mechanism in LUAD remains poorly studied. ETS1 is an important transcription factor that regulates matrix metalloproteinases [40]. ETS1 also mediates cell migration, angiogenesis, and drug resistance [41,42]. Recently, increasing attention has been paid to the regulatory role of ETS1 in the energy metabolism of tumor cells. For example, ETS1 promotes the aggressiveness of LUAD cells in different ways [43,44]. Therefore, our data combined with previous studies suggest that circ_0007618 and However, there were certain limitations to these analyses. First, all patients were selected from the same hospital, and therefore, this study was a cross-sectional observational study. Our conclusions should be validated within different ethnic groups in different regions. Second, the circRNAs and screened core genes should be further validated in cell experiments, including a sufficient luciferase assay.

## Conclusions

circ_0007618 and circ_0029426 were selected via bioinformatic analyses to participate in the LUAD process by regulating PIK3CA, NRAS, KRAS, and ETS1, and the combination of these two indicators may assist in the diagnosis and prognosis of LUAD.

## Supplementary Material

Supplemental Material

## Research Highlights

- circ_0007618 and circ_0029426 were revealed to be significantly dysregulated in LUAD.
- A circRNA–miRNA–mRNA network was established for LUAD diagnosis.
- The combination of circ_0007618 and circ_0029426 is a potential biomarker for LUAD diagnosis and prognosis.
- PIK3CA and NRAS, and KRAS and ETS1, were targeted by circ_0007618 and circ_0029426, respectively.
