# Supplementary figures and images for: Circular RNA–microRNA–mRNA network identified circ_0007618 and circ_0029426 as new valuable biomarkers for lung adenocarcinoma
Source: Bioengineered. 2022 Feb 25;13(3):6258–71. doi: 10.1080/21655979.2022.2027180 (PMC8973644; doi:10.1080/21655979.2022.2027180)

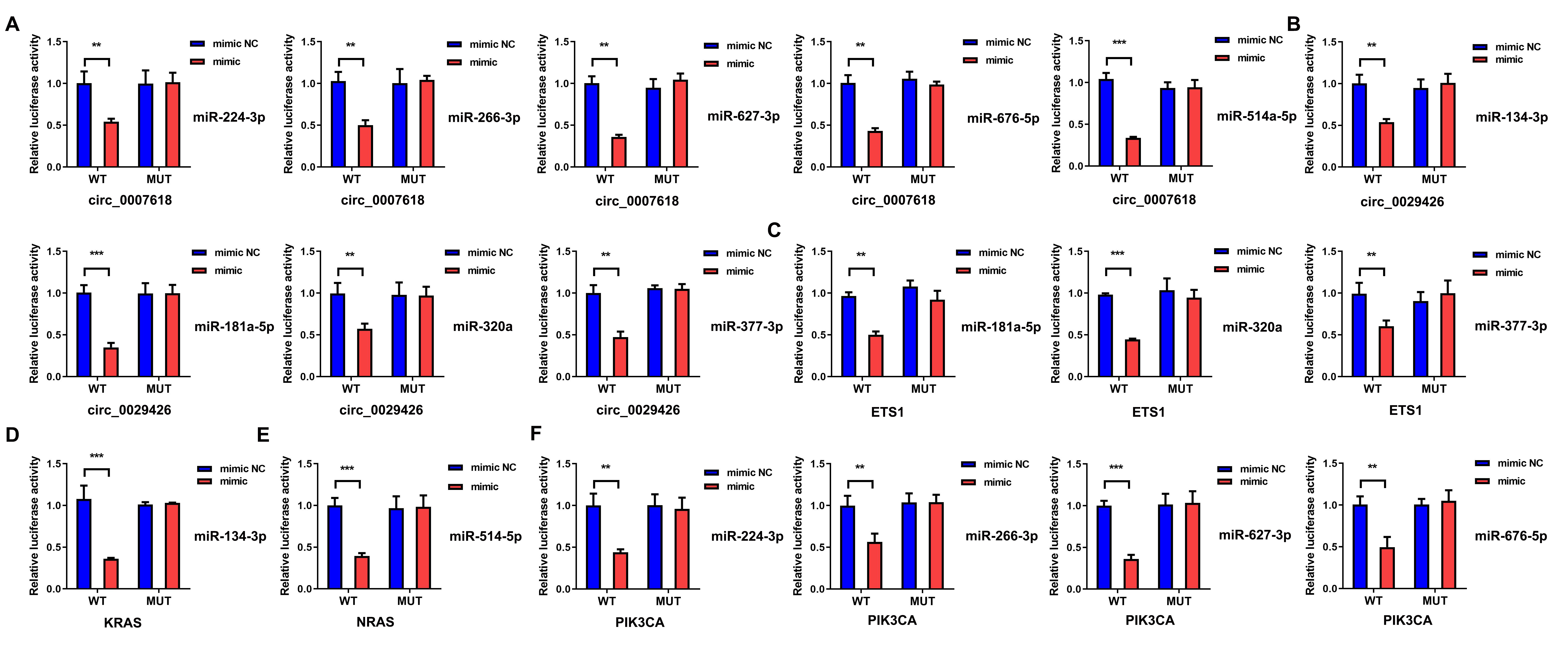

Supplement: Supplemental Material [file KBIE_A_2027180_SM4604.zip › supplementary/S1.jpg]

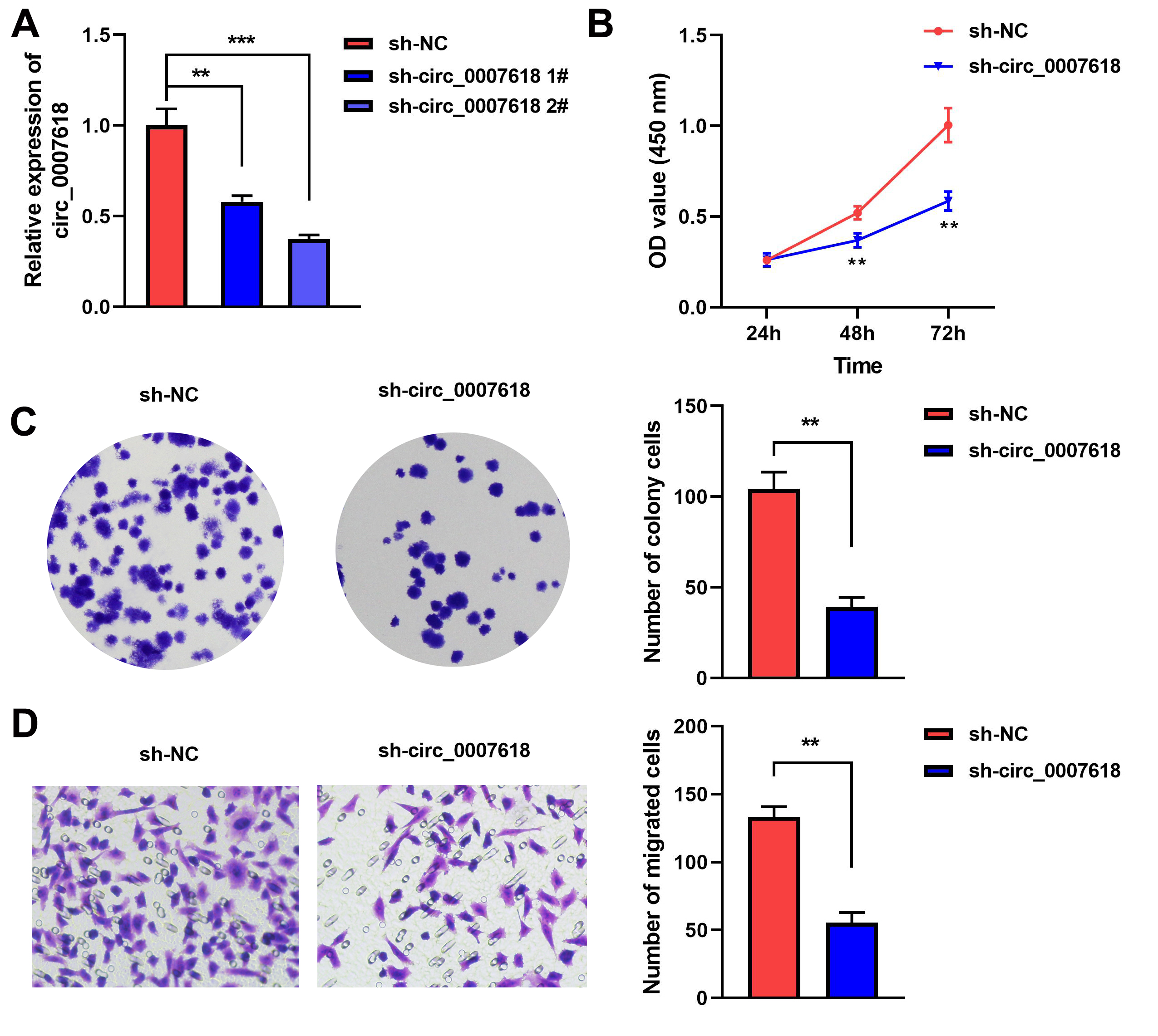

Supplement: Supplemental Material [file KBIE_A_2027180_SM4604.zip › supplementary/S2.jpg]
